# Initiation of *cyp26a1* Expression in the Zebrafish Anterior Neural Plate by a Novel Cis-Acting Element

**DOI:** 10.1371/journal.pone.0150639

**Published:** 2016-03-09

**Authors:** Chunhong Chen, Aline Stedman, Emmanuelle Havis, Isabelle Anselme, Daria Onichtchouk, François Giudicelli, Sylvie Schneider-Maunoury

**Affiliations:** 1 CNRS, UMR 7622, Paris, France; 2 Sorbonne Universités, Université Pierre et Marie Curie, Institut de Biologie Paris-Seine (IBPS), Developmental Biology Laboratory, Paris, France; 3 Inserm, U1156, Paris, France; 4 Developmental Biology, Institute Biology I, Faculty of Biology, University of Freiburg, Freiburg, Germany; Deakin School of Medicine, AUSTRALIA

## Abstract

Early patterning of the vertebrate neural plate involves a complex hierarchy of inductive interactions orchestrated by signalling molecules and their antagonists. The morphogen retinoic acid, together with the Cyp26 enzymes which degrade it, play a central role in this process. The *cyp26a1* gene expressed in the anterior neural plate thus contributes to the fine modulation of the rostrocaudal retinoic acid gradient. Despite this important role of *cyp26a1* in early brain formation, the mechanisms that control its expression in the anterior neural plate are totally unknown. Here, we present the isolation of a 310-base-pair DNA element adjacent to *cyp26a1* promoter, displaying enhancer activity restricted to the anterior neural plate of the zebrafish gastrula. We show that unlike that of *cyp26a1*, expression driven by this *cyp26a1* anterior neural plate element (cANE) is independent of retinoic acid. Through deletion analysis, we identify a 12-nucleotide motif essential for cANE activity. A consensus bipartite binding site for SoxB:Oct transcription factors overlaps with this motif. Mutational analysis suggests that SoxB binding is essential for its activity. We discuss the contribution of this study to the elucidation of the regulatory hierarchy involved in early neural plate patterning.

## Introduction

During neural induction, the embryonic neural plate becomes gradually regionalized along its antero-posterior (AP) axis. Signals from adjacent tissues control the regionalised expression in the neural plate of a set of transcription factors, which translate this positional information into specific developmental programs. The AP pattern is later refined under the influence of secondary organizing centers. This patterning process is essential to elaborate the diverse neural regions and cell types forming the CNS. Three main signalling pathways, the Wnt, Fgf and retinoic acid (RA) pathways, cooperate to pattern the neural plate along the AP axis [1].

*cyp26a1* encodes a retinoic acid degrading enzyme of the cytochrome p450 family [2]. *cyp26a1* orthologues have been found in all vertebrate species analysed (reviewed in [3]). In zebrafish, three *cyp26* genes, *cyp26a1*, *b1* and *c1*, are expressed in the neural plate and act partially redundantly in hindbrain patterning by regulating the spatio-temporal gradient of RA [4]. *cyp26a1* arises first, at late blastula stages, and is the only *cyp26* gene expressed anterior to the hindbrain. This high anterior expression is roughly complementary to that of *hoxb1b* [1,5,6]. At the early somite stages, *cyp26a1* starts to be expressed in an additional, more caudal domain in the hindbrain, in an anterior-to-posterior decreasing gradient.

Functional studies in several vertebrate species have identified three distinct functions for Cyp26a1 in the neural plate, all concurring to the fine modulation of RA signalling. First, in the anterior neural plate, where the gene is expressed at high levels, Cyp26a1 directly protects from the posteriorizing influence of RA, via its degradation. Second, the anterior RA sink resulting from this degradation generates a decreasing caudorostral gradient of RA across the hindbrain. Third, direct upregulation of *cyp26a1* expression by RA in the hindbrain creates a negative feedback loop that scales the RA gradient, reducing its sensitivity to variations in RA global amount and contributing to the coordination of patterning with embryo elongation. In support of this working model[4,6], zebrafish *cyp26a1* mutants display defects reminiscent of excess RA, including expansion of posterior structures into anterior hindbrain territory [7] and show uniform concentration of RA along the AP axis [8]. Moreover, exposure of these embryos to non teratogenic doses of exogenous RA leads to a massive posterior transformation of the whole anterior neural plate [4], suggesting that Cyp26a1 activity protects the anterior neural plate from RA teratogenicity. Mouse *Cyp26a1* knock-out embryos die at mid-gestation and display similar hindbrain posteriorization defects [9,10].

*cyp26a1* expression is RA-inducible and, consistently, the *cyp26a1* promoter contains RA-responsive elements. However, while low-level *cyp26a1* expression in the hindbrain is upregulated by exposure to RA, high-level *cyp26a1* expression in the anterior neural plate does not require RA signalling [1,6]. Moreover, while the above model requires that the anterior RA sink be set up independently of RA itself, how this is achieved in terms of initiation and maintenance of *cyp26a1* expression in the anterior neural plate remains unknown. Roles for the transcription factors TGIF, Zic1 and B1 Sox (Sox1/2/3/19) have been proposed [11–13]. *tgif* knock-down in zebrafish embryos results in a reduction in both levels and extent of *cyp26a1* expression during gastrulation, while *tgif* overexpression by mRNA injection is able to activate *cyp26a1* expression ectopically, likely via indirect regulation since TGIF has solely been characterized as a transcriptional repressor [11]. *Zic1* knock-down leads to a reduction of *cyp26a1* expression in the forebrain, while midbrain expression is maintained [12]. It is not known whether the activity of Zic1 on *cyp26a1* expression is direct. Finally, knockdown of all four B1 *sox* genes (*sox2*/*3*/*19a*/*19b*) in zebrafish shows that these genes are essential for neural development and, in particular, for neural expression of *cyp26a1*. Potential B1 Sox binding sites were found in the *cyp26a1* promoter. Moreover, ChIP analyses and luciferase assays pointed to a direct role of B1 Sox transcription factors in *cyp26a1* activation [13]. Interestingly, both TGIF and Zic factors have been associated to holoprosencephaly, the most common human congenital forebrain defect [14]. In both cases, it has been proposed that the defects may arise from deficient degradation of RA in the forebrain, perhaps resulting from deficient Cyp26 function [11,12]. Despite these important results, the regulatory sequences that drive *cyp26a1* expression in the anterior neural plate remain to be identified.

In this paper we describe a novel regulatory element, close to the promoter of zebrafish *cyp26a1*, which is able to drive GFP reporter gene expression in the anterior neural plate during gastrulation with a pattern very similar to that of endogenous *cyp26a1* expression. We use this element to query the mechanisms that govern the initiation of *cyp26a1* activation in the neural plate. This element displays properties markedly distinct from previously characterized cis-regulatory elements of *cyp26a1*, in that it is activated independently of RA, rather depending on the activity of early expressed Sox transcription factors. We show that these properties unexpectedly reside in the least evolutionarily conserved part of the element.

## Results and Discussion

### Identification of a neural plate-specific *cyp26a1* enhancer

**Fig 1:**
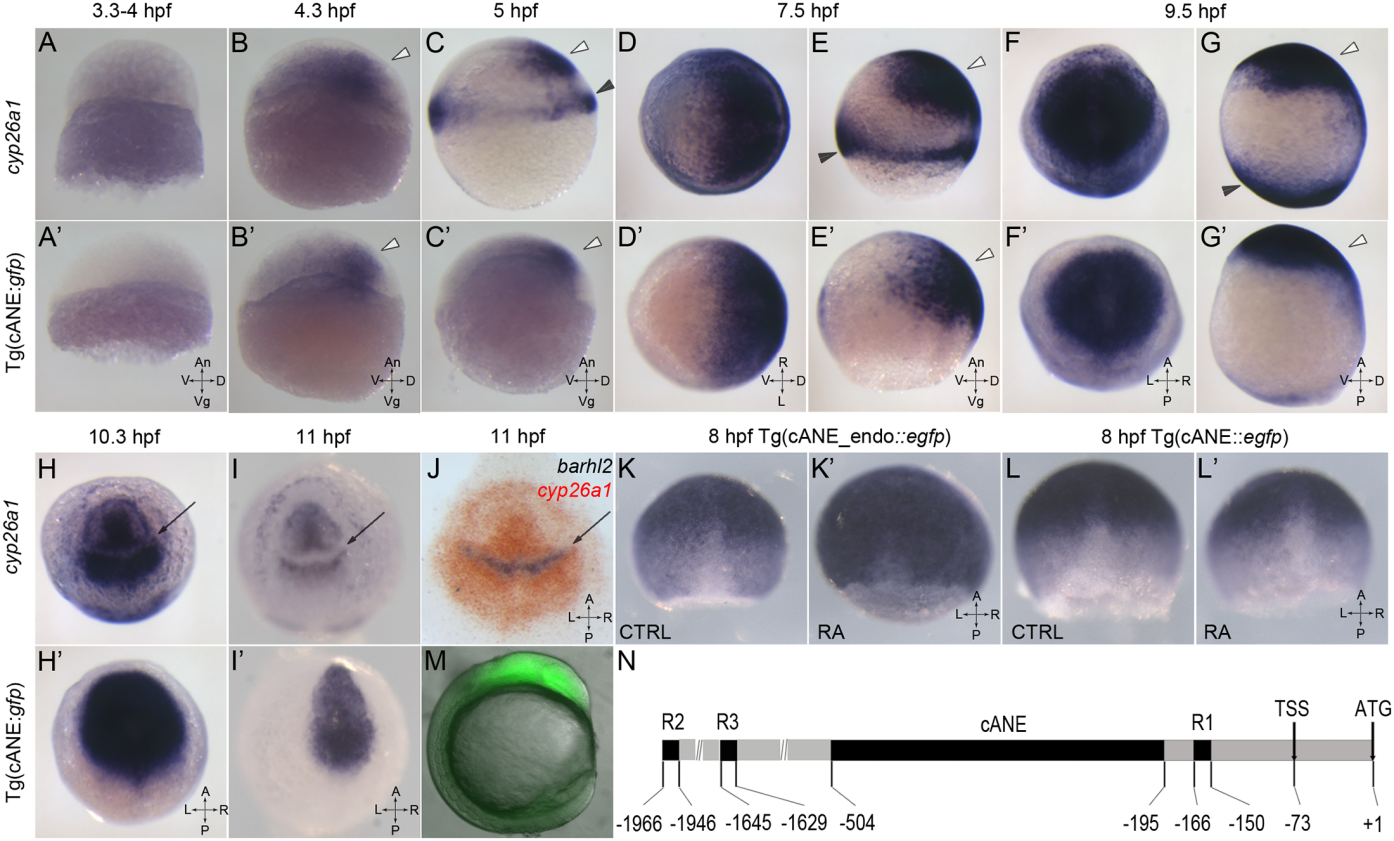
Characterization of cANE activity as an early neural plate specific enhancer. (A-I,A’-I’) Compared expression patterns of *cyp26a1* (A-I) and *egfp* driven by cANE (A’-I’) during early embryonic development. (J) Double in situ hybridization showing *barhl2* expression domain (blue) exactly filling the gap in the *cyp26a1* expression domain (red). (A,B,C,E,G,A’,B’,C’,E’,G’) are lateral views. (d,f,h,I,D’,f’,h’,I’) are animal pole views. White arrowheads: anterior neural plate. Black arrowheads: blastoderm marginal zone. Arrows in (H-J): gap in the anterior neural plate domain of *cyp26a1* expression. All stages are indicated in the pictures. (k,k’,l,l’) The effect of 100 nM retinoic acid (RA) treatment between 2,5 hpf and 8,5 hpf on on stable transgenic cANE_endo:::*egfp* (K-K’) and cANE::*egfp* (L-L’) expression. (M) EGFP fluorescence in a 12 hpf stable transgenic cANE:::*egfp* embryo. Lateral view with dorsal to the left. (N) Schematic representation of cANE and all three reported retinoic acid responsive elements (R1, R2, R3) identified previously. cANE is located from -504 bp to -195 bp relative to *cyp26a1* ATG codon. An: animal, Vg: vegetal; V: ventral; D: dorsal, A: anterior, P: posterior, L: left, R: right, CTRL: control embryo, RA: retinoic-acid treated embryo.

In the course of a chromatin immunoprecipitation (ChIP) experiment aimed at identifying regulatory elements bound by the Irx7 transcription factor in the neural plate of gastrulating zebrafish embryos, we recovered a 310 bp element, located in the immediate upstream vicinity of the promoter of the cyp26a1 gene. When cloned upstream of a minimal *gata2* promoter driving expression of *egfp* (Tol2::gata2::*egfp* vector) [15] and injected into fertilized zebrafish eggs, this reporter construct yielded consistent and robust expression of *egfp* in the anterior neural plate at 8–10 hours post fertilization (hpf). To confirm the enhancer activity of this element, we generated a transgenic line of zebrafish, which had stably incorporated the construct in their genome. EGFP fluorescence in live transgenic embryos was conspicuous in the anterior part of the head as early as 12 hpf (Fig 1M). Therefore, the 310 bp element harbors a transcriptional enhancer activity that is reminiscent of *cyp26a1* expression in the anterior neural plate. For this reason, we named it cyp26a1 Anterior Neural Element (hereafter cANE). Even though we were not able to determine whether our initial recovery of cANE was due to Irx7 regulating *cyp26a1*, the important role of Cyp26 enzymes in early neural plate patterning, combined to the relative lack of data about early *cyp26a1* regulation, prompted us to study cANE in detail.

We carefully examined the spatio-temporal expression pattern of the cANE-controlled transgene by in situ hybridization with an *egfp* antisense probe, and compared it with the expression pattern of the *cyp26a1* gene (Fig 1). The earliest cANE::*egfp* expression could be observed at dome stage (4.3 hpf), and coincided with the activation of *cyp26a1* (Fig 1A, 1A’, 1B and 1B’). Similarity between the two was most obvious between shield (5–6 hpf) and early somite (11 hpf) stages in the neural plate (Fig 1B–1I’). During this period, cANE::*egfp* was expressed in a single domain corresponding precisely to the anterior neural plate domain of *cyp26a1* expression (white arrowhead in Fig 1B, 1B’, 1C, 1C’, 1E, 1E’, 1G and 1G’). In contrast, cANE::*egfp* was never seen in the distinct *cyp26a1* expression domain at the blastoderm margin (black arrowheads in Fig 1C, 1E and 1G; compare with Fig 1C’, 1E’ and 1G’). This expression domain closely resembles that of the earliest known genes expressed in the anterior neural plate, such as *sox2* [16].

As somitogenesis began (10 hpf onwards), the first divergence between neural plate expression of *cyp26a1* and cANE::*egfp* appeared: while *cyp26a1* expression was downregulated in a transverse domain in the prospective brain, generating a gap between two domains of roughly equivalent size in the anterior and posterior brain (arrow in Fig 1H, 1I and 1J), cANE::*egfp* remained expressed in the whole region, including the *cyp26a1*-negative domain (Fig 1h’ and 1I’). Since this gap in *cyp26a1* forebrain expression had not been described before, (although it is visible in Fig 1G of [4]), we performed double in situ hybridisation with *cyp26a1* and anterior diencephalic marker *barhl2* [17], and demonstrated that the gap corresponded precisely to the anterior part of the prospective diencephalon, since *barhl2* and *cyp26a1* expression domains were perfectly complementary (Fig 1J).

Hence, cANE is an anterior neural plate-specific enhancer of *cyp26a1*, which recapitulates its expression between 4 and 10 hpf, although it potentially lacks a cis-repressor acting in the anterior part of presumptive diencephalon after 10 hpf. Thus, cANE is one of the most early-expressed region-specific enhancers in the neural plate. Its activation must therefore involve primordial mechanisms of neural plate regionalisation.

### The *cyp26a1* anterior neural plate enhancer is independent of RA

**Fig 2:**
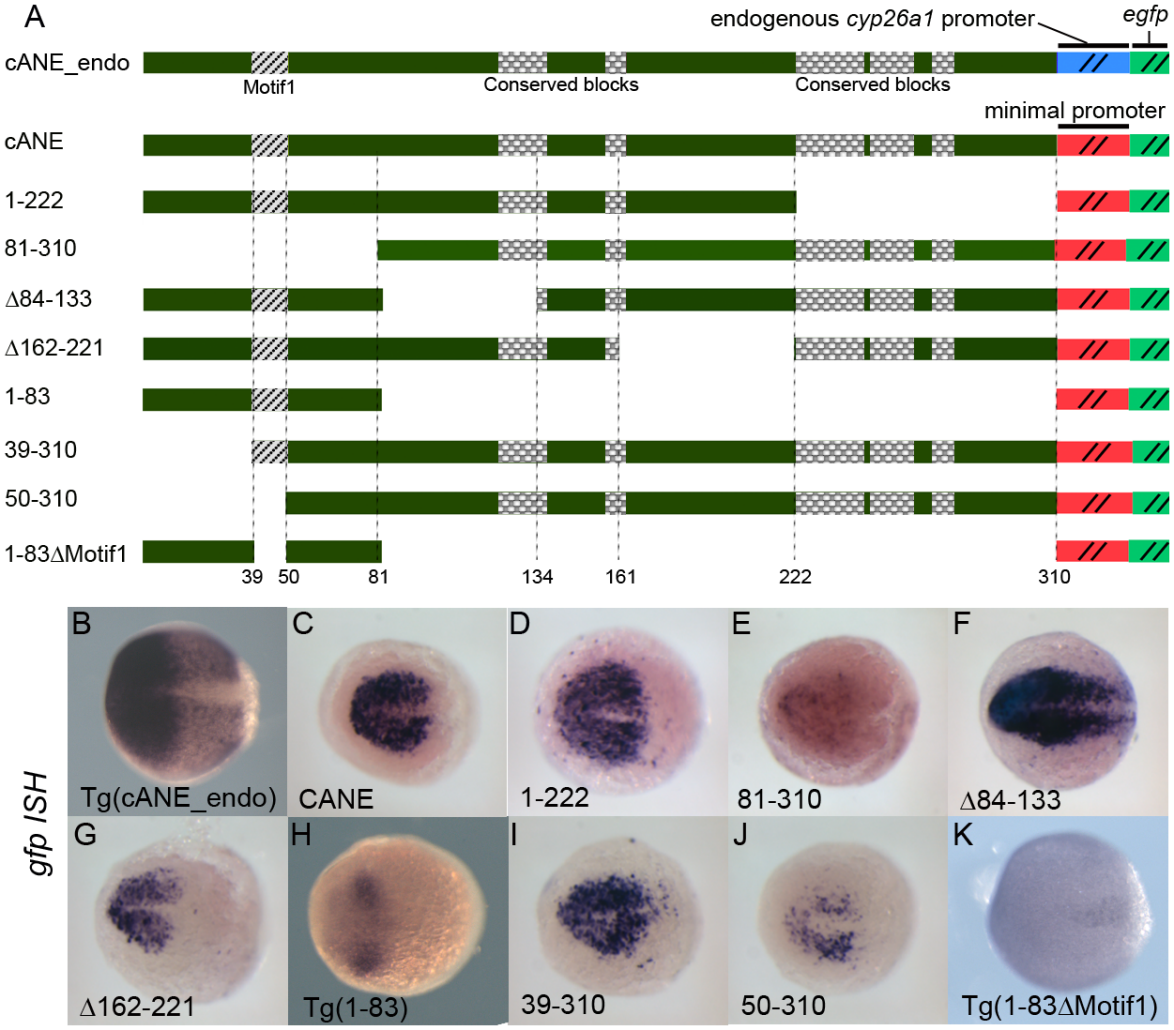
Detailed dissection of cANE by transient expression of deletion constructs in zebrafish embryos. (A) Schematic view of the constructs used for dissection of the cANE module. All constructs contain one single fragment (black) from the cANE module, placed immediately upstream of the *gata2* minimal promoter, except for construct cANE_endo, where the *gata2* promoter is replaced by the *cyp26a1* endogenous promoter. Numbers correspond to nucleotide coordinates within the 310 nt zebrafish cANE. The hatched block (Motif1) represents the 12-bp difference between constructs 39–310 and 50–310; the highly conserved blocks are indicated by checkered patterns. (B-K) Enhancer activity of the cANE deletions shown in (A), assayed by *egfp* in situ hybridization in transient or stable (labelled Tg()) transgenic embryos. All embryos are between stages 10.5 and 11 hpf, viewed from the animal pole, with anterior on the left.

In order to compare the specific enhancer activity of cANE with that of more proximal promoter elements, we produced a transgenic line, cANE-endo::*egfp*, in which *egfp* expression was driven by cANE cloned upstream of the endogenous *cyp26a1* promoter (nucleotides -195 to +1 relative to the translation initiation codon) instead of the minimal *gata2* promoter (Figs 1N and 2A). In addition to the anterior neural plate, these additional regulatory elements led to expression of *egfp* in 2 other *cyp26a1*-expressing tissues, the presumptive hindbrain (Figs 1K and 2B) and the blastoderm margin (not shown).

Similar to other *cyp26* genes, *cyp26a1* is a known target of retinoic acid (RA) signalling [1,2,5,18]. In the zebrafish anterior neural plate in particular, raising RA levels results in a caudal expansion of *cyp26a1* expression [1]. Accordingly, several RA response elements (RARE)s that drive expression in the neural plate have been identified in the *cyp26a1* promoter [18–21]. We reasoned that the activity of cANE-endo::*egfp* in the hindbrain may be due to the presence of previously characterized RA response elements lying next to cANE, in *cyp26a1* promoter region (Fig 1N). This raised the possibility that cANE might represent a novel, RA-independent, anterior neural plate enhancer. To test this possibility, we treated transgenic cANE::*egfp* and cANE-endo::*egfp* embryos with RA from 3 to 8 hpf. Although the expression of *cyp26a1* itself was strongly enhanced by RA treatment (data not shown), we observed no increase in the expression of cANE::*egfp*; instead, cANE::*egfp* was slightly but reproducibly downregulated in RA-treated embryos (Fig 1L and 1L’). In contrast, expression of cANE-endo::*egfp* was upregulated (Fig 1K’), consistent with the presence of an RA-responsive element in the *cyp26a1* promoter.

Therefore, expression of cANE in the anterior neural plate is activated neither directly nor indirectly by RA. This contrasts with all previously characterized regulatory regions of *cyp26a1*, including the 200 bp immediately upstream of the ATG, which positively and strongly respond to RA [20] (and Fig 1K’). Theoretical work has clearly highlighted that proper shaping of the rostrocaudal RA gradient required some kind of RA-independent upregulation of *cyp26a1* in the anterior neural plate in order to form a high cyp26 domain acting as a RA sink [6]. Hence, the present cANE materializes a previously predicted cis-acting regulatory element. The observed downregulation of cANE::*egfp* upon RA treatment is reminiscent of that of *otx2* expression in similar conditions [1] and may be attributed to indirect repression of anterior neural plate gene expression by RA.

### Deletion analysis identifies a 12 bp motif essential for cANE activity

In order to identify functional elements responsible for cANE activity, we first undertook an unbiased deletion analysis. We split the cANE into four parts of equal size and designed a corresponding set of four deletions to yield reporter constructs 81–310::*egfp*, Δ84–133::*egfp*; Δ162–221::*egfp* and 1–222::*egfp*, respectively (Fig 2A). These four deletions were tested for enhancer activity in transient and/or germline transgenesis using the Tol2_gata2::*egfp* reporter construct (Fig 2).

Embryos expressing 1–222::*egfp* showed exactly the same *egfp* expression as cANE::*egfp* (Fig 2B and 2D). Thus the 89 bp 3’ extremity is not necessary for full cANE activity in the ANP. In contrast, the three other reporters led to *egfp* expression patterns markedly different from that of cANE::*egfp* (Fig 2B, 2E, 2F and 2G) in transient transgenesis experiments. Δ84–133::*egfp* had stronger *egfp* expression in the ANP as well as ectopic expression posterior to the midbrain (Fig 2F), suggesting the existence of negative regulatory elements inside the 84–133 fragment, required to confine cANE activity inside the ANP. Meanwhile, Δ162–221::*egfp* showed *egfp* expression only in the anteriormost part of the ANP (Fig 2G) meaning that the 162–221 fragment is required for cANE activity in the posterior part of ANP (mid/hindbrain). Finally, 81–310::*egfp* exhibited only very weak residual expression of *egfp* (Fig 2E), indicating that fragment 1–80 is essential for cANE activity. Conversely, a 1–83::*egfp* construct was sufficient to drive *egfp* expression in the ANP, both in transient transgenesis (not shown) and in transgenic lines, albeit at lower levels and in a smaller territory than full cANE (Fig 2H). The 1–83 fragment thus contains regulatory elements important for proper expression in the ANP. In order to narrow down the location of these elements, we performed additional deletions of cANE distal-most region, 39–310::*egfp* and 50–310::*egfp* (Fig 2A) and tested them in transient transgenesis. 39–310::*egfp* showed no overt difference with cANE::*egfp* (Fig 2I). In contrast, 50–310::*egfp* showed greatly decreased *egfp* expression (Fig 2J). Since these two constructs differ by 12 nucleotides only, we reasoned that this 12-nucleotide Motif1 (hatched box in Fig 2A) must contain important information for cANE activity. Accordingly, deletion of Motif1 from the 1–83 fragment (in the 1–83ΔMotif1::*egfp* transgenic line, Fig 2) resulted in loss of *egfp* expression in the anterior neural plate (Fig 2K).

### Conserved regions inside the cANE are not essential for its activity

**Fig 3:**
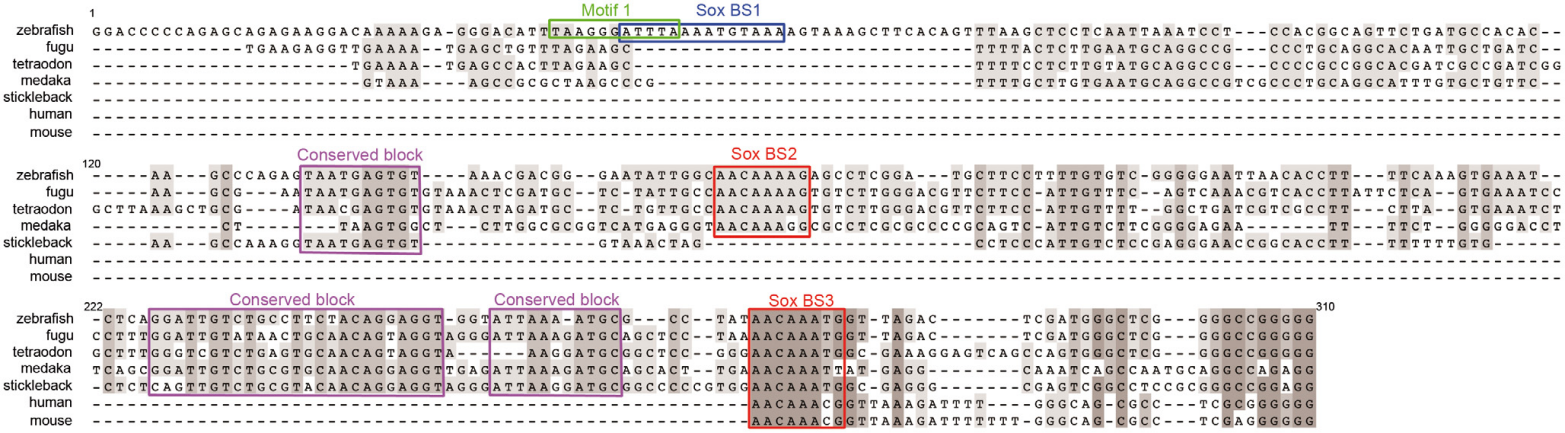
Conservation of vertebrate *cyp26a1* promoter region with zebrafish cANE. Alignment of 4 teleostean (tetraodon, fugu, stickleback and medaka) and mouse and human *cyp26a1* promoter regions orthologous to zebrafish cANE. The most conserved regions are outlined with red (predicted SoxB binding sites 2 and 3) or purple (conserved block1,2,3) boxes. The green (Motif1) and blue (predicted SoxB binding site 1) boxed sequences lie in the non-evolutionarily conserved region of zebrafish cANE. Numbers correspond to nucleotide coordinates within the 310 nt zebrafish cANE.

The early anterior neural plate expression of *cyp26a1* has been conserved between at least mouse [22,23] and zebrafish [1] during evolution from their common ancestor, and therefore likely in most other Euteleostomi species. Since cis-acting regulatory elements of genes with evolutionarily conserved expression profiles generally tend to be conserved [24], we examined conservation of *cyp26a1* promoter regions among several species. The available UCSC genome browser alignment of the zebrafish genome (http://genome.ucsc.edu/) identifies regions homologous to zebrafish cANE in the *cyp26a1* promoter regions of four fish genomes: medaka, fugu, tetraodon and stickleback (Fig 3). Moreover, the proximal-most region also shows conservation with mammalian genomes (mouse and human, Fig 3). In addition, two of these conserved regions contain motifs (red boxes in Fig 3) resembling the consensus binding site [25] for the HMG type transcription factors Sox of the B1 group (Sox2/3/19a/19b), anterior neural plate determinants expressed at very early stages of neural plate formation [26,27]. Strikingly, the most conserved region of cANE maps to the 222–310 fragment that is dispensable for enhancer activity in the ANP (it is deleted in 1–222::*egfp*, which reproduces full cANE activity). In contrast, the Motif1, identified in our deletion analysis as essential for zebrafish cANE activity, is not conserved (Fig 3). This finding conflicts with the typical tendency for regulatory motifs to be evolutionarily conserved, supporting instead several published indications that regulatory elements underlying conserved gene expression patterns need not necessarily show primary sequence conservation [28–30].

### B1 Sox transcription factors are candidate positive regulators of cANE activity

**Fig 4:**
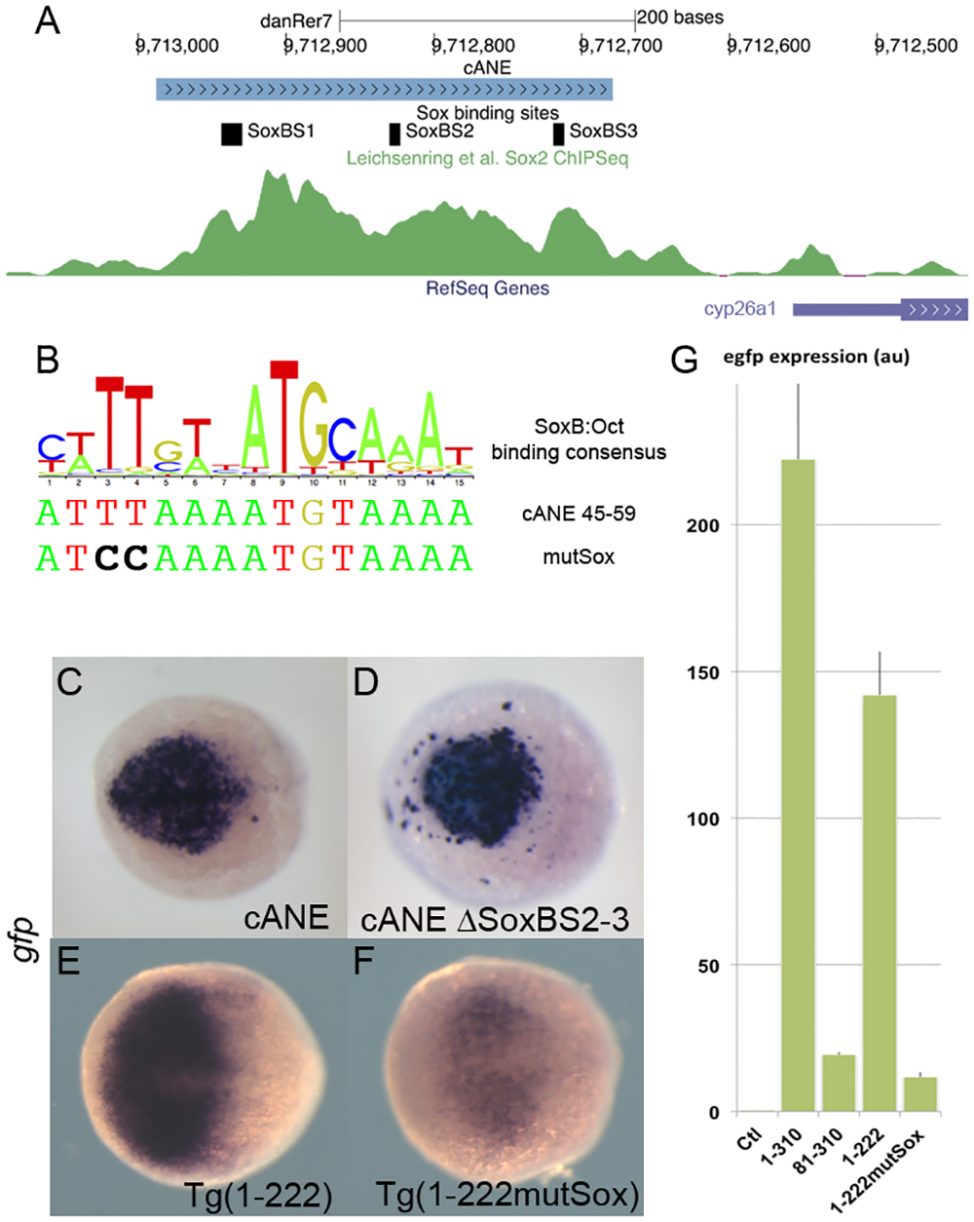
Role of SoxB binding sites in the regulation of *cyp26a1* expression in the anterior neural plate (ANP). (A) Representation of the cANE region in the zebrafish genome; 3 predicted SoxB binding sites are indicated; the green channel represents the intensity of the anti-Sox2 ChipSeq signal in this region according to [34]. (B) A logo representing the composite consensus binding site for SoxB/Oct factors [33] is aligned with the predicted Sox binding site (Sox_BS1) overlapping Motif1 (cANE 45–59). The mutSox TT->CC mutation destroying the Sox-binding half-site is indicated. (C-F) egfp in situ hybridization representing enhancer activity of cANE ΔSoxBS2-3, where both Sox_BS2 and Sox_BS3 have been deleted (C), compared with intact cANE (D), at the 90% epiboly stage, and enhancer activity of Motif1 mutation mutSox (F) in 1–222 context compared with wild type 1–222 (E), at the 75% epiboly stage. Dorsal views; anterior is to the left. (G) RT-PCR relative quantification of total *egfp* expression stable transgenic embryos for constructs cANE (1–310), 81–310, 1–222 and 1–222 mutSox, as well as non transgenic embryos (Ctl); error bars represent SEM; units are arbitrary.

Scanning cANE for putative transcription factor binding sites with the Genomatix MatInspector tool [31] and the Jaspar database scanning tool [32] highlighted three sequences matching binding sites for B1 Sox transcription factors (Figs 3 and 4A). Within them, a sequence, that significantly matches the composite binding motif for Sox:Oct dimers [33], overlaps with Motif1 (Fig 3).

Several recent published results point at a function of B1 group Sox factors in the activation of *cyp26a1*. First *cyp26a1* expression in the ANP is eliminated in zebrafish embryos depleted of all four B1 group Sox factors (Sox2/3/19a/19b) [13]. Then, anti-Sox2 ChIP-PCR amplifies a 167 bp fragment of the *cyp26a1* upstream sequences [13] and this fragment overlaps the last 89 bp of cANE (nucleotides 222–310 in Fig 3), suggesting that Sox2 binds cANE in vivo. Furthermore, in an unbiased ChIP-Seq study, Sox2 was abundantly associated, in late blastula embryos, to a 500 bp region encompassing cANE, just after the onset of zygotic transcription [34] (Fig 4A). Finally, knock-down of the four B1 Sox genes leads to downregulation of a luciferase reporter construct containing 1.6kb of *cyp26a1* promoter [13], including cANE.

Hence, we tested the role of the three putative Sox binding sites (SoxBS), pink boxes in Fig 4A) present inside cANE. We first tested the role of the two other putative SoxBS identified in cANE. When SoxBS2 and SoxBS3 were both deleted, the resulting reporter construct, ΔSoxBS2-3::*egfp* showed no overt difference with cANE::*egfp*, in its ability to drive reporter expression in the anterior neural plate when transiently expressed in embryos (Fig 4D). This means that these two predicted SoxBSs are not required for cANE activity. Thus, SoxBS1 is the best candidate site to mediate the previously demonstrated action of the B1 Sox family on *cyp26a1* expression.

To test the importance of the putative SoxB:Oct binding site (SoxBS1), we generated a mutation in the core of the predicted BS (Fig 4A). The mutation was introduced in the Sox-binding half-site (AT**TT**AAATGTAAA changed to AT**CC**AAATGTAAA) in 1–222::*egfp* (resulting in 1-222MutSox::*egfp* construct). 1-222MutSox::*egfp* showed expression decreased both in staining intensity and in the extent of the expression territory compared to intact 1-222::*egfp*, both in transient expression experiments (not shown) and in stable transgenic lines (Fig 4E and 4F). Real-time RT-PCR quantification of total *egfp* expression in stable transgenic embryos at 90% epiboly stage revealed that, while cANE:::*egfp* and 1–222:::*egfp* expressed roughly equivalent amounts of *egfp* transcripts, this amount was reduced by one order of magnitude in both 81–310:::*egfp* and 1-222MutSox:::*egfp* (Fig 4G).

Thus, we identified a 12 bp-cis-acting regulatory region, Motif 1, required for cANE activity. Mutation of two nucleotides in a site within Motif1 that matches a consensus SoxB binding site strongly reduces cANE activity. Together with data from the literature, this strongly suggests that B1 Sox factors activate *cyp26a1* expression via binding Motif1. These data therefore provide a potential mechanism for *cyp26a1* activation in the anterior neural plate.

### Conclusion

We have identified a *cyp26a1* anterior neural plate enhancer that it is independent of RA and dependent on a B1 Sox transcription factor binding site. Despite *cyp26a1* expression in the anterior neural plate being conserved among vertebrate species, the DNA motifs we identified as important for cANE activity suprisingly do not appear to be evolutionarily conserved, while the conserved domains do not appear important for proper expression. This should therefore call for caution when considering the relationships between sequence conservation and function. In order to determine whether, beyond sequence, the molecular cues involved in *cyp26a1* expression are evolutionary conserved, it will be necessary to identify a functional cANE element in other vertebrate species.

This characterization of cANE as one of the earliest regulatory element acting in the anterior neural plate constitutes an advance toward elucidating how the regulation of the *cyp26a1* gene is achieved in order to shape the rostrocaudal gradient of RA that later patterns the vertebrate brain.

## Materials and Methods

### Constructs

All constructs used were based on Tol2_gata2::egfp reporter plasmids [35], with candidate regulatory elements inserted in the PstI/XhoI restriction sites in front of the minimal gata2 promoter. cANE deletions were engineered by standard molecular biology protocols, mainly by PCR-based mutagenesis. All the PstI/XhoI inserts were sequence-verified. cANE_endo construct was generated by replacing the *gata2* minimal promoter in cANE:::*egfp* by the zebrafish *cyp26a1* promoter sequence, in continuity with cANE and extending up to the translation initiation codon.

### Zebrafish strains

Zebrafish were raised and maintained as described previously [36]. Embryos were staged according to the number of hours postfertilization (hpf) at 28°C.

All animal manipulations complied to European directive 2010/63, under the control of the veterinary services of Paris (authorization #75–419).

### DNA and mRNA injection

For transient transgenesis, eggs were injected at the 1-cell-stage with approximately 1 nl of a solution containing 25 ng/μl plasmid DNA and 25 ng/μl in vitro transcribed Tol2 transposase mRNA [35].

For each construct, at least 3 separate injection experiments were performed, and at least 20 embryos from each experiment were processed through in situ hybridization and imaged. Although the precise expression patterns of *egfp* could somewhat vary from one embryo to the other, as it commonly happens in transient transgenesis, the embryos chosen to illustrate Figs 3 and 4 were all chosen as the most representative of their kind.

Stable transgenic lines, designated ‘Tg(…)’ in the figures, were generated by raising injected fish to adulthood and screening them by EGFP fluorescence at 24hpf for transmission of the transgene to their progeny.

### In situ hybridization

In situ hybridization was performed as described previously [37]. Probes used were *egfp* (antisense to the mRNA encoded by the Tol2_gata2::egfp plasmid [35]), *cyp26a1* [4], *barhl2* [17].

### Retinoic acid treatment

All-trans RA (Sigma R2625) was added to the embryo medium at early blastula stage (2.5 hpf), with a final concentration of 100 nM. Treated and control embryos were fixed 6 h later, when approaching completion of epiboly, then processed for in situ hybridization.

### RT-PCR

For quantitation of *egfp* expression, total RNA was extracted from batches of 50 embryos at 90% epiboly stage, using Trizol (Thermo Fisher Scientific). cDNA first strand was synthetized using Superscript III Reverse Transcriptase (Thermo Fisher Scientific) with random priming. Quantitative PCR was performed on a Bio-Rad CFX96 system with SYBR Green. *egfp* was amplified with primers TATATCATGGCCGACAAGCA (forward) and ACTGGGTGCTCAGGTAGTGG (reverse). For normalization, quantification of *ef1alpha* was used with the same primer pair as described in [38]. All samples were run in triplicates. For each sample, *egfp* normalized expression was computed by dividing measured *egfp* expression by *ef1alpha* expression.
